# Significance of Matrix Metalloproteinase 9 Expression as Supporting Marker to Cytokeratin 19 mRNA in Sentinel Lymph Nodes in Breast Cancer Patients

**DOI:** 10.3390/ijms17040571

**Published:** 2016-04-21

**Authors:** Marek Murawski, Marta Woźniak, Kamila Duś-Szachniewicz, Paweł Kołodziej, Marta Rzeszutko, Piotr Ziółkowski

**Affiliations:** 1 Department of Gynecology and Obstetrics, Wrocław Medical University, Chałubińskiego 3, 50-368 Wrocław, Poland; marcopolo2135@gmail.com; 2 Department of Pathology, Wrocław Medical University, Marcinkowskiego 1, 50-368 Wrocław, Poland; marta1wozniak@wp.pl (M.W.); kamila.dus@gmail.com (K.D.-S.); rzemarta@wp.pl (M.R.); 3 Division of Pathology, Sokołowski Hospital Wałbrzych, Sokołowskiego 4, 58-309 Wałbrzych, Poland; hp2010@wp.pl

**Keywords:** invasive breast carcinoma of no special type, one-step nucleic acid amplification, cytokeratin 19, matrix metalloproteinase 9

## Abstract

One-step nucleic acid amplification (OSNA) detects and quantifies, with the use of a polymerase chain reaction, the presence of cytokeratin 19 mRNA in sentinel lymph nodes. The main advantage of the OSNA assay is the avoidance of second surgery in case of positive sentinel lymph node diagnosis. The objective of this study was to evaluate the significance of matrix metalloproteinase 9 expression by immunohistochemistry as supporting marker to cytokeratin 19 mRNA in sentinel lymph nodes in breast cancer patients and to relate this expression with clinicopathological data. This study was conducted on fresh sentinel lymph nodes obtained from 40 patients with tumors classified as carcinoma of no special type. The presence of metastatic cells in the slices of lymph nodes was evaluated by immunohistochemistry using antibodies for CK19 and MMP-9. Expression of CK19 and MMP-9 in lymph nodes was also confirmed by means of Western blot analysis. Results indicated that the strongest correlation with CK19 mRNA was displayed by MMP-9, CK19 (by immunohistochemistry, IHC), and nodal metastases (*p* < 0.001). Higher histological grading also positively correlated with CK19 mRNA, however that correlation was less significant. Since MMP-9 shows very strong correlation with CK19 mRNA in breast carcinoma of no special type metastases, expression of MMP-9 in sentinel lymph nodes should be considered as useful method whenever OSNA analysis is not available.

## 1. Introduction

The evaluation of sentinel lymph node (SLN) status is a primary procedure of breast cancer stage assessment in patients with clinically negative axilla [1]. Previous studies have suggested that patients with micrometastases in SLNs can avoid total lymphadenectomy [2]. Selective SLN biopsy has replaced axillary lymph node dissection thanks to its reduced morbidity and promising prognosis [3,4]. Nodal metastases are very important prognostic factor and they can help in the assessing the need for adjuvant therapy.

A number of different methods has been used to examine SLN status in the past studies. Most often employed was the standard histological evaluation during routine microscopic procedure on formalin-fixed and paraffin-embedded tissues [5]. This procedure seems very practical, economic, reliable, and can be supplemented by other methods including immunohistochemical stainings. An important and obvious limitation of this method is that patients with positive SLN are subjected to additional surgical procedure [6]. Another method of evaluating SLN is intraoperatory histological examination of frozen SLN [6] but it is, in turn, limited by, among other factors, relatively low accuracy.

All above mentioned methods also have other limitations, such as the study using only a part of the lymph node, due to slice cutting on a microtome.

Another approach is the search for a molecular fingerprint as a substitute marker of metastatic cancer cells. Previous studies indicated the use of cytokeratin 19. When these molecular analyses were compared to morphologic study of SLN, their sensitivity exceeded 90% with almost perfect specificity. An assay called one-step nucleic acid amplification (OSNA) was then proposed, validated, and standardized in the studies of SLN in patients with the breast cancer. OSNA detects and quantifies, with the use of a polymerase chain reaction (PCR) the presence of mRNA of cytokeratin 19 in the lymph node [7]. It is more time-consuming than the routine hematoxylin-eosin study, but it can be done during surgical operation [8,9].

The main advantage of the OSNA assay is the avoidance of additional surgery in case of patients with positive SLN diagnosis. In addition to the positive impact on the quality of life of patients, OSNA assay provides a measurable economical profit by reducing the use of the surgical room and decreasing extra costs of hospitalization [10,11]. Avoiding second surgery also allows quicker introduction of necessary adjuvant therapy in patients with positive SLN. This is a major issue since the introduction of adjuvant therapy before 12 weeks after surgery has often been reported to increase recurrence-free and overall survival in early-stage breast cancer [12] and overall survival in metastatic breast cancer patients [13]. Another advantage of OSNA is its quantitative nature, providing the means to assess whether there exists a threshold of high risk of metastasis that would justify an axillary lymph node (ALN) resection and adjuvant treatment with all associated morbidities [13]. The American Society of Breast Surgeons reported, that new molecular techniques based upon the analysis of the whole SLN could be predictive of non-SLN metastases and thresholding CK19 mRNA copy numbers could provide a rational way for avoidance of ALN resection in patients with one or two positive SLNs subjected to tangential radiotherapy [14].

Matrix metalloproteinases (MMPs), a family of zinc-dependent endopeptidases, are found in cellular milieu of various tissues, including malignant ones like e.g., breast carcinoma, and they are involved in degradation of extracellular matrix. Findings suggested that MMPs overexpression might indicate a higher probability of poor prognosis in breast cancer. This especially refers to MMP-9 high levels in serum. Among the matrix metalloproteinases, MMP-9, and also MMP-2, which are highly expressed in various malignant tumors, are crucial in the degradation of type IV collagen, and are considered to be associated with invasion and migration of malignant cells. MMP-9 and MMP-2 are upregulated in all human and animal tumors and their expression is increasing with each and every stage of tumor progression [15]. MMP-9 showed a strong immunostaining in patients with metastasis that had died, whereas there was no expression of this marker in normal breast tissues [16]. Some previous studies reported independently that breast cancer patients with high serum or tumor MMP-9 expression showed a poor prognosis [17,18,19,20,21].

The objective of this study was to evaluate the significance of matrix metalloproteinase 9 expression by immunohistochemistry as supporting marker to cytokeratin 19 mRNA in sentinel lymph nodes in breast cancer patients, and to relate this expression with clinicopathological data (staging including nodal metastases confirmed by CK19 immunoreactivity, histological grading, and age of patients) because of their prognostic value and receptor expression (ER, PR, HER2) in primary tumors because of their predictive significance. Analysis of expression of CK19 mRNA and MMP-9 was performed on sentinel lymph node by OSNA and immunohistochemistry, respectively.

Our results showed that the strongest correlation with CK19 mRNA was displayed by MMP-9, CK19, and nodal metastases. Higher histological grading also positively correlated with CK19 mRNA, however that correlation was less significant.

## 2. Results

Table 1 shows the data from IHC studies together with CK19 mRNA, staging, and histological grading (G) established for each patient in the whole group. It has to be emphasized that neither immunohistochemical nor Western blot analysis revealed any expression of matrix metalloproteinase-2 (MMP-2) in tissues from SLNs. Therefore, we did not show the relevant data in figures and table.

### 2.1. Analysis of Sentinel Lymph Nodes by Means of One-Step Nucleic Acid Amplification (OSNA)

#### 2.1.1. Correlation of CK19 mRNA with CK19 and MMP-9 by Means of Immunohistochemistry (IHC)

The incidence of lymph node metastases in the OSNA group was 17/40 (42.5%). In the conventional CK19 IHC group metastases were found in 9/40 cases (22.5%). The correlation between CK19 mRNA and MMP-9 was very strong, *i.e.*, in 17/40 *vs.* 15/40 cases, respectively (*p* < 0.001), the expression was found to be positive, whereas the correlation between metastases confirmed by CK19 (IHC) and MMP-9 was weaker, *i.e.*, in 9/40 *vs.* 15/40 cases (*p* > 0.05). Table 2 shows correlations between three markers and metastases in lymph nodes.

Our results confirmed significant correlation between 3 studied markers and nodal status (*p* < 0.05).

#### 2.1.2. Correlation of CK19 mRNA with Clinicopathological Data

Correlation between grading of primary breast carcinoma and three markers, CK19 mRNA, CK19 (by IHC), and MMP-9 is shown in Table 3. CK19 mRNA and MMP-9 were significantly more often expressed in patients with G2 and G3 cancers (*p* < 0.01) in comparison with G1 tumors.

Correlation between positive expression of estrogen, progesterone, and HER2 expression, and three markers: CK19 mRNA, CK19 (by IHC), and MMP-9, is shown in Table 4. Estrogen was positive in 14, while progesterone was positive in 13 cases of CK19 mRNA-positive SLNs. Estrogen was in turn, positive in only seven and progesterone in six cases of CK19-positive SLNs, by means of IHC. Higher number of cases expressed MMP-9, *i.e.*, 12 for positive estrogen and 11 for progesterone positive cases. In our study, HER2 was negative in 15 cases of CK19 mRNA-positive SLNs, and positive in only two. HER2 was negative in eight cases of CK19-positive SLNs by means of IHC (only one case was HER2 positive), while HER2 was negative in 13 cases of MMP-9-positive SLNs, and in two—was positive.

No significant correlation was found between CK19 mRNA, CK19 (by IHC), and MMP-9, and status of hormone and HER2 receptors (*p* > 0.05).

The age of patients ranged from 37 to 82 (mean: 59.05) and the largest group of patients was between 51 and 60. OSNA confirmed metastases in six (age 51–60 and 61–70), two (41–50), and three (71–80). MMP-9 was expressed in two of two OSNA SLN positive patients (age 41–50 and 71–80), five of six (51–60), and six of six (61–70). The age of patients correlated positively with OSNA results, however it was not significant (*p* > 0.05), (see Table 5).

Correlation between Spearman’s coefficient of correlation and Pearson χ-square values for CK19 mRNA and MMP-9, and analyzed clinicopathological features are shown in Table 5.

The strongest correlation with CK19 mRNA were observed for MMP-9 (ρ = 0.898), CK19 (ρ = 0.625), and nodal metastases (*N*) (ρ = 0.614). Positive correlation between above features was significant (*p* < 0.001). Histological grading (*G*) also positively correlated with CK19 mRNA, however that correlation was less significant (ρ = 0.387; *p* < 0.05).

The tumor status (*T*) correlated positively, however it was not significant (*p* > 0.05).

The degree of compliance diagnoses between CK19 mRNA and MMP-9 or CK19 (by IHC) showed that Cohen’s kappa coefficient was 0.896 for CK19 mRNA and MMP-9, *i.e.*, indicated almost perfect agreement, whereas it was only 0.564 for CK19 mRNA and CK19 (by IHC) and indicated moderate agreement.

### 2.2. Immunohistochemistry and Western Blot Analysis

**Figure 1:**
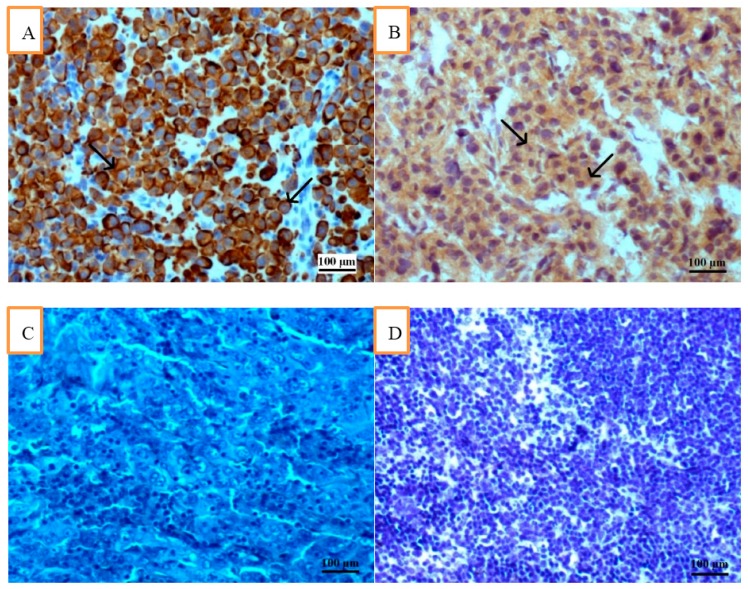
(**A**) Sentinel lymph node from the patient with metastatic breast invasive carcinoma NST G2. Immunohistochemical staining for cytokeratin 19 (streptavidin-biotin method, hematoxylin counterstained, 100×). Positive reactions have been observed in metastatic carcinoma cells (arrows); (**B**) Sentinel lymph node from the patient (same as in Figure 1A) with metastatic breast invasive carcinoma NST G2. Immunohistochemical staining for matrix metalloproteinase-9 (streptavidin-biotin method, hematoxylin counterstained, 100×). Positive reactions have been observed in cytoplasm of metastatic carcinoma cells (arrows); (**C**) Negative control. Sentinel lymph node from the patient with metastatic breast invasive carcinoma NST G2. No immunostaining was found. First antibody was omitted in this case; hematoxylin, 100×; (**D**) Negative control in normal lymph node from breast cancer-free patient. Staining for matrix metalloproteinase-9 (streptavidin-biotin method, hematoxylin counterstained, 100×) revealed no reactivity in this case.

Figure 1A shows the result of staining for cytokeratin 19 in SLN with metastasis of breast carcinoma of NST (by means of IHC), whereas Figure 1B—for MMP-9.

**Figure 2:**
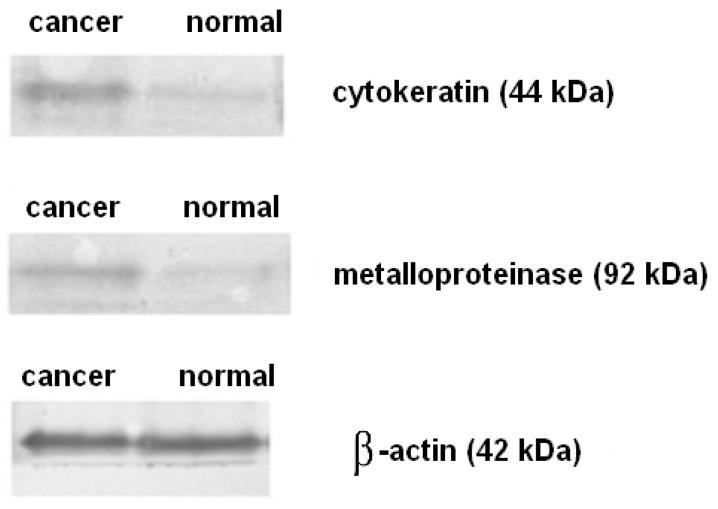
Western blot analysis of sentinel lymph node from the patient with metastatic breast invasive carcinoma NST G2 (same patient as in Figure 1). **Left** lane shows bands of cancer tissue while **right** lane shows bands of normal breast tissue. β-Actin was used as a control protein.

Figure 2 shows Western blot for CK19 and for MMP-9 of sentinel lymph node from the patient with metastatic breast invasive carcinoma NST G2. It can be seen clearly that the protein level of cytokeratin 19 and metalloproteinase 9 was substantially higher than that of normal tissue.

## 3. Discussion

Previous studies have reported that the classical histopathological analysis of SLNs significantly underestimates the axillary staging of breast carcinoma in comparison with molecular studies of SLNs by OSNA [8,22,23]. Ruano *et al.* [24] confirmed this finding in a large study comparing the use of OSNA and histopathology for staging of axillary lymph nodes in breast cancer patients with positive SLNs. Accordingly to OSNA, standard routine histopathological analysis misclassified almost 42% of patients as negative for axillary node metastasis. Ruano *et al.* [24] also confirmed that whole lymph node examination by means of OSNA assay detects more metastases than standard histopathological study. These results were consistent with data reported by Santaballa *et al.* [22].

Increased number of lymph nodes with micrometastases provides information that is hard to apply in clinical settings because the prognostic value of this finding is not well clarified. In one prospective trial, comparing outcomes of SLN biopsy alone with axillary dissection, more than 5000 patients with SLN biopsy were involved in the study [4] and the presence of occult metastases was found to be associated with a lower overall survival, progression free survival, and distant metastases-free interval compared to node-negative patients. Despite of above increased risk in the three parameters, authors concluded that the differences might be of little clinical relevance. Notwithstanding, the analysis of the whole lymph node by means of OSNA allows to perform lymphadenectomy during the same operation in all patients, which is not always possible in cases studied by means of IHC in which the complete results could be obtained later. Important advantages for the patient resulting from a single procedure comprise avoidance of second surgery and a possible delay in the start of adjuvant therapy. Another advantage are lower institutional costs [10].

When using the OSNA assay, more micrometastases can be found than by means of standard histopathological studies [8]. The OSNA assay may improve the accuracy and standardized evaluation of the residual tumor burden after neoadjuvant chemotherapy, even when chemotherapy-induced histopathological changes are present [25]. The main cause of discordant results may be tissue allocation bias.

Ohi *et al*. [26] showed that the tumor size, angioinvasion and CK19 mRNA copy number were independent predictors of non-SLN involvement. The CK19 mRNA copy number had a high odds ratio. The tumor size in the SLN was most often considered as an important predictive factor for non-SLN involvement. However, these standard histopathological examinations evaluating the size of metastases are prone to interobserver variability and usually have limited ability for accurate detection of the metastatic volume in nodes, because observations are made on only a part of the lymph node. It has been stated that the OSNA assay has an almost equivalent reliability as routine histopathological examinations for the prediction of non-SLN metastases. Tamaki *et al.* [23] proved also that OSNA assay can be used for routine clinical SLN biopsy and its assessment for volume of metastasis may be a powerful predictive factor for non-SLN metastasis. In our study we have observed significant increase in the number of positive lymph nodes, *i.e.*, expressing CK19 mRNA on the OSNA analysis in comparison to metastases found in standard immunohistochemistry, *i.e.*, from nine to 17 in 40 examined cases.

Previously, the MMP-9 showed a strong immunostaining in deceased breast cancer patients with metastasis, whereas there was no marker in normal breast tissues [16]. High levels of MMP-2 and MMP-9 in serum were strongly connected with lymph node metastasis and, additionally, activities of MMP-2 and MMP-9 were significantly increased in metastatic than in non-metastatic lymph nodes [27]. Authors concluded that serum MMP-9 levels may have a diagnostic value for predicting axillary node metastasis [27]. In 2005 Decock *et al.* [28] found that MMP-9 levels did not correlate with any of the investigated variables, like nodal status or with any of the classical clinicopathological factors including histological tumor type, tumor size, and grade and hormone receptor status. Our results showed that MMP-9 also correlated less significantly with the above factors (rho below 0.5 and *p* > 0.05 except for nodal status, histological grading and CK19 by IHC).

Previous study showed that MMP-9 protein was primarily found in the cytoplasm of both cancer and stromal cells [29] and, in another study, its expression in matched epithelium and lymph node was associated with lymph node metastasis [30]. Interestingly, in another study correlation of MMP-9 expression with clinicopathological characteristics, including histological type, tumor size, grade and ER, PR and HER2 status was not found [30]. By contrast, in another study the tissue and serum levels of MMP-9 were found to be associated with histology grade, lymph node status, pathological stage, lymphovascular invasion, and also associated with ER [31]. Sullu *et al.* [32] reported that expression of MMP-9 was increased in triple-negative, high-grade, and ER-negative cancers and cancers with distant metastases. Expression of MMP-9 was also increased in cases with overexpression of HER2, however no statistically significant difference was found. No correlation was observed between lymph node metastasis or tumor size and expression of MMP-9 [32]. Our findings showed that SLNs positive for MMP-9 were negative for ER in only three cases (20% of all MMP-9 positive SLNs) and they were found in three of six triple negative cases (50%).

Huang *et al.* [33] reported that, in comparison to low expression, high serum and tissue levels of MMP-2 were connected with metastases in lymph nodes and higher TNM stage, high tissue levels of MMP-9 were connected with metastases in lymph nodes and higher TNM stage, as well as high serum levels of MMP-9 were connected with expression of c-erbB-2. In our study we did not observe statistically significant correlation of MMP-9 with the size of primary breast tumors (*T*).

In summary, our results indicated that the strongest correlation with CK19 mRNA was displayed by MMP-9, CK19 (by IHC), and nodal metastases. Positive correlation between above features was significant (*p* < 0.001). Higher histological grading, G2 and G3, also positively correlated with CK19 mRNA, however that correlation was less significant. Since MMP-9 shows very strong correlation with CK19 mRNA in breast carcinoma of no special type metastases, immunohistochemical expression of MMP-9 in SLNs should be considered as a useful method whenever the OSNA analysis for some reason is not physically available or it is not recommended for patients previously subjected to breast surgery because the CK19 expressed in epidermal cells migrates then to the inside of the breast tissues and may be detected by the OSNA assay and interpreted as a false positive [10]. For this reason, a combination of two or three markers should be recommended. Additionally, the immunohistochemistry is still less expensive and widely used in different laboratories. Most laboratories are equipped with autostainers dedicated to IHC while they cannot afford to purchase more expensive devices. Therefore, we would strongly suggest using MMP-9 by IHC when OSNA is, for the above reasons, not available.

## 4. Materials and Methods

### 4.1. Patients

The study was reviewed and approved by the Ethics Committee of the Wrocław Medical University (17/2015) and conducted in accordance to Declaration of Helsinki of 1975, revised in 2008. Written informed consent was obtained from all patients before surgical procedures.

The study was conducted on fresh SLNs sampled from 40 consecutive female patients bearing a tumor with a maximum diameter not greater than 3 cm, and with physically non-palpable axillary lymph nodes between 2014 and 2015. Histologically, tumors were classified as carcinoma of no special type (formerly invasive ductal carcinoma).

Patients were subjected to breast-conserving surgery or to modified radical mastectomy and, in cases where the OSNA assay was positive, axillary lymph node dissection was performed in the same session. Altogether, we tested SLNs from 40 patients with carcinoma of no special type (grade 1, 2, or 3). Cancers were graded according to Bloom and Richardson and the staging according to the Unione Internationale Contre le Cancer tumor-node-metastasis (TNM) system criteria [34].

### 4.2. Sentinel Lymph Node Sampling Method

SLNs were identified using three technetium 99 m-labeled, nano-sized, human serum albumin colloid subcutaneous injections around the nipple. To avoid any contamination during operation, lymph nodes were surgically excised before the lumpectomy and immediately sent on ice to the Department of Pathology. SLNs were cut into numerous slices which were then processed by the OSNA method or were fixed with neutral buffered formaldehyde and embedded in paraffin for further routine staining with hematoxylin and eosin (HE).

### 4.3. One-Step Nucleic Acid Amplification (OSNA)

OSNA assay was carried out according to the manufacturer’s instructions (Sysmex, Kobe, Japan). Briefly, tissue slices cut from the lymph nodes were homogenized on ice in 4 mL of the Lynorhag homogenizing buffer (Sysmex). A small aliquot was then used for automated real-time amplification of CK19 mRNA via reverse transcription loop-mediated isothermal amplification with the ready-to-use Lynoamp Kit (Sysmex) on the RD-100i (Sysmex). The degree of amplification was detected via a by-product of the reaction, *i.e.*, pyrophosphate. The excess lysate was stored at −80 °C after use. A CK19 mRNA copy number/µL lysate less than 250 was regarded as negative (−) and above 250 as positive (+), thus indicating lymph node status: negative or micrometastasis, respectively. Negative and positive controls of CK-19 were included in each analysis.

### 4.4. Immunohistochemistry (IHC)

For immunohistochemistry 5 µm thick sections were cut from the slices of the all 40 SLNs, and they were immunostained with anti-CK19 mAb IR615 (Dako, Glostrup, Denmark) diluted 1:50 and with anti-MMP-2 (Abcam, Cambridge, UK, ab37150) and anti-MMP-9 (Abcam, ab58803), both diluted 1:100. The presence of metastatic cells in the slices of SLNs was evaluated by IHC using anti-CK19 and the SLNs were also stained for MMP-2 and MMP-9 using anti-MMP-2 and anti-MMP-9 antibodies. Immunoreactions were revealed by a streptavidin-biotin enhanced immunoperoxidase technique in an Automated Stainer Link 48 and EnVision™ Flex Kit (Dako, Denmark). When more than 15% of cells expressed staining for MMP-2, MMP-9, and CK19 the reaction was considered as positive (+); otherwise it was considered as negative (−). Our series of primary breast tumors were tested for estrogen (ER) and progesterone (PR) expression using monoclonal antibodies from Dako, Denmark and for HER2 overexpression using the polyclonal antibody A0485 (Dako). HER2 reaction by means of IHC was determined according to ASCO/CAP guidelines [35] as: 0 and 1+ negative, 2+ equivocal, and 3+ positive. ER and PR were determined according to the Allred scoring system [36] when showing distinct nuclear immunoreactivity. When HER2 was found to be (2+)-equivocal, HER2 FISH was carried out. Evaluation of the IHC results, blinded to all patient data, was performed independently by two pathologists. Discrepancies in evaluation were resolved by means of a joint viewing of the slides under a multiheaded microscope.

### 4.5. Western Blotting Analysis

For Western blotting analysis, appropriate tissue samples from formalin-fixed paraffin-embedded blocks were macrodissected. Sections were deparaffinized and treated with lysis buffer (4% SDS, 0.1 M DTT, in 0.1 M Tris/HCl buffer pH 7.6) containing Protease and Phosphatase Inhibitor Cocktails (Sigma Aldrich, Taufkirchen, Germany). After homogenization, samples were lysed in Thermomixer R (Eppendorf, Hamburg, Germany) with an agitation for 1 h. Next, protein extracts were centrifuged at 16,000× *g* at 25 °C for 15 min. The protein concentration in the supernatant was measured at 280 nm using a NanoDrop 2000 spectrophotometer (Thermo Fisher Scientific, Inc., Waltham, MA, USA). Proteins were separated on 4%–12% SDS-PAGE electrophoresis (sodium dodecyl sulfate polyacrylamide gel electrophoresis, equipment from Invitrogen, Carlsbad, CA, USA) and transferred to the nitrocellulose membrane (GE Healthcare, Little Chalfont, UK). The membrane was blocked with 10% goat serum (Sigma-Aldrich) in PBS with 0.1% Tween for 30 min at room temperature. Subsequently, the membranes were immunoblotted overnight at 4 °C with the primary antibodies against matrix metalloproteinase 9 that recognizes both the latent ~92 kDa and the active ~83 kDa (dilution 1:100, Merck Millipore, Darmstadt, Germany), cytokeratin 19 (dilution 1:100, Santa Cruz, Biotechnology, Inc., Heidelberg, Germany). The next day, membranes were washed 2 × 5 min with PBS, and incubated with horseradish peroxidase-labeled secondary goat anti-rabbit antibody (dilution 1:2000, Santa Cruz Biotechnology, Inc.) for 1 h at room temperature and, thereafter, washed 3 × 5 min with PBS. The protein bands were visualized by reaction with DAB using a DAB Enhanced Liquid Substrate System for Immunochemistry (Sigma Aldrich) and the results were documented using Molecular Imager Gel Doc TMXR+ (Bio-Rad, Hercules, CA, USA). Loading differences were normalized by using a monoclonal β-actin antibody against the housekeeping control β-actin (dilution 1:1000, Abcam).

### 4.6. Statistics

Associations between the various markers and clinicopathological data were analyzed using the Pearson Chi-square test and Spearman’s Rank Coefficient. The Cohen’s *κ* coefficient yields a measure of the level of agreement among CK19 mRNA, CK19 (by IHC), and MMP-9. The study used a significance level of 0.05, *i.e.*, results were considered to be statistically significant at *p* < 0.05. The software used for the analysis was STATISTICA v.10 (StatSoft, Inc., Tulsa, OK, USA).

The degree of compliance diagnoses:

- *κ*-Values between 0.41 and 0.60 indicated moderate agreement,
- *κ*-Values between 0.61 and 0.80 indicated substantial agreement,
- *κ*-Values above 0.81 indicated almost perfect agreement.

## 5. Conclusions

The matrix metalloproteinase-9 showed very strong correlation with CK19 mRNA in breast carcinoma of no special type metastases, and therefore immunohistochemical expression of this marker in SLNs should be considered as a useful method whenever the OSNA analysis for some reason is not physically available or it is not recommended for patients previously subjected to breast surgery. The combination of two or three markers, like cytokeratin 19 and matrix metalloproteinase-9 should then be recommended.

## Figures and Tables

**Table 1:** Clinico-pathological characteristics of 40 patients with breast carcinoma of no special type, and relevant immunohistochemical and CK19 mRNA data. In each of 40 cancer cases only one lymph node has been examined. CK19 mRNA (OSNA), CK19, and MMP-9 (IHC) were evaluated in SLNs, whereas receptors in primary tumors. HER2 (+)—means 3+ or 2+ and after FISH verified as 3+ (positive); N0—no macrometastasis has been found; N1-3—macrometastasis in lymph node has been found; pTNM—established before OSNA analysis; L (LV)—lymphatic vessels embolism; B (BV)—blood vessels embolism; in each case the lymphatic and blood vessels were free of invasion.

No.	Age	Receptors	CK19 mRNA	CK19	MMP-9	Staging pTNM	*G*
1	71	ER(3+) PR(−) HER2(+)	+	+	+	T2N1aMXL0B0	3
2	53	ER(1+) PR(3+) HER2(−)	+	−	+	T1N0MXL0B0	1
3	49	ER(1+) PR(2+) HER2(−)	−	−	−	T1cN0MXL0B0	1
4	45	ER(1+) PR(2+) HER2(−)	+	+	+	T1bN2aMXL0B0	3
5	57	ER(3+) PR(3+) HER2(−)	−	−	−	T1cN0MXL0B0	3
6	43	ER(2+) PR(2+) HER2(+)	−	−	−	T1cN0MXL0B0	2
7	54	ER(3+) PR(−) HER2(−)	−	−	−	T1aN0MXL0B0	1
8	37	ER(−) PR(−) HER2(−)	−	−	−	T1cN0MXL0B0	3
9	64	ER(3+) PR(2+) HER2(+)	+	−	+	T1cN0MXL0B0	3
10	68	ER(1+) PR(+) HER2(−)	+	+	+	T2N1aMXL0B0	2
11	82	ER(3+) PR(3+) HER2(−)	−	−	−	T2N0MXL0B0	3
12	66	ER(−) PR(−) HER2(−)	−	−	−	T1cN0MXL0B0	1
13	51	ER(1+) PR(3+) HER2(−)	−	−	−	T1cN0MXL0B0	3
14	57	ER(2+) PR(3+) HER2(−)	+	+	+	T1bN3aMXL0B0	2
15	74	ER(−) PR(−) HER2(+)	−	−	−	T1N0MXL0B0	2
16	54	ER(2+) PR(−) HER2(−)	−	−	−	T1cN0MXL0B0	3
17	40	ER(−) PR(−) HER2(−)	−	−	−	T1cN0MXL0B0	1
18	67	ER(3+) PR(3+) HER2(−)	−	−	−	T2N0MXL0B0	1
19	59	ER(1+) PR(3+) HER2(−)	+	−	+	T1cN0MXL0B0	3
20	59	ER(−) PR(−) HER2(+)	−	−	−	T2N0MXL0B0	3
21	70	ER(3+) PR(3+) HER2(−)	+	−	+	T1cN0MXL0B0	2
22	38	ER(1+) PR(3+) HER2(−)	−	−	−	T1cN0MXL0B0	1
23	57	ER(3+) PR(2+) HER2(−)	+	+	−	T1bN1aMXL0B0	1
24	65	ER(3+) PR(3+) HER2(−)	−	−	−	T1bN0MXL0B0	1
25	79	ER(2+) PR(3+) HER2(−)	+	−	+	T2N0MXL0B0	2
26	46	ER(1+) PR(1+) HER2(+)	−	−	−	T1N0MXL0B0	1
27	52	ER(3+) PR(2+) HER2(−)	−	−	−	T1cN0MXL0B0	1
28	57	ER(3+) PR(2+) HER2(−)	−	−	−	T1cN0MXL0B0	2
29	62	ER(3+) PR(2+) HER2(+)	−	−	−	T1bN0MXL0B0	1
30	62	ER(3+) PR(3+) HER2(−)	+	+	+	T2N1MXL0B0	2
31	66	ER(3+) PR(−) HER2(+)	−	−	−	T2N0MXL0B0	1
32	59	ER(3+) PR(3+)HER2(−)	+	−	+	T1bN0MXL0B0	3
33	67	ER(3+) PR(3+) HER2(−)	−	−	−	T2N0MXL0B0	1
34	76	ER(2+) PR(3+) HER2(−)	+	+	−	T2N1MXL0B0	2
35	56	ER(1+) PR(1+) HER2(−)	−	−	−	T1bN0MXL0B0	1
36	70	ER(−) PR(−) HER2(+)	−	−	−	T1aN0MXL0B0	1
37	67	ER(2+) PR(1+) HER2(−)	+	−	+	T1N0MXL0B0	2
38	63	ER(−) PR(−) HER2(−)	+	−	+	T2N0MXL0B0	3
39	54	ER(−) PR(−) HER2(−)	+	+	+	T1N1MXL0B0	3
40	46	ER(−) PR(−) HER2(−)	+	+	+	T1aN1MXL0B0	2

**Table 2:** Correlation between expression of CK19 mRNA, CK19 (by IHC) and MMP-9, and nodal status.

Positive Expression	N0 *n* = 31	>N0 *n* = 9	Fisher’s Exact Test	Accuracy
CK19 mRNA	8 (25.8%)	9 (100.0%)	*p* < 0.001	80%
CK19 (by IHC)	0 (0.0%)	9 (100.0%)	*p* < 0.001	100%
MMP-9	8 (25.8%)	7 (71.4%)	*p* = 0.002	75%

**Table 3:** Correlation between grading of primary breast carcinoma and three markers: CK19 mRNA, CK19 (by IHC), and MMP-9.

Positive Expression	Grade 1 *n* = 16	Grade 2 *n* = 11	Grade 3 *n* = 13	Pearson χ-Square	Spearman’s Coefficient of Correlation
CK19 mRNA	2 (12.5%)	8 (72.7%)	7 (53.8%)	*p* = 0.005	ρ = 0.385
CK19 (by IHC)	1 (6.2%)	5 (45.4%)	3 (23.1%)	*p* = 0.056	ρ = 0.199
MMP-9	1 (6.2%)	7 (63.6%)	7 (53.8%)	*p* = 0.003	ρ = 0.443

**Table 4:** Correlation between positive expression of estrogen, progesterone, and HER2, and three markers: CK19 mRNA, CK19 (by IHC), and MMP-9. In all groups *p* > 0.05.

Positive Expression	Estrogen *n* = 31	Progesterone *n* = 27	HER2 *n* = 9
CK19 mRNA	14 (45.2%)	13 (48.2%)	2 (22.2%)
CK19 (by IHC)	7 (22.6%)	6 (22.2%)	1 (11.1%)
MMP-9	12 (38.7%)	11 (40.7%)	2 (22.2%)

**Table 5:** Correlation between Spearman’s coefficient of correlation and Pearson χ-square values for CK19 mRNA and MMP-9, and analyzed clinicopathological features.

Variable	CK19 mRNA		MMP-9	
	ρ	*p*	ρ	*p*
Age	0.253	0.114	0.159	0.321
ER	−0.033	0.838	−0.045	0.781
PR	0.090	0.575	0.120	0.454
HER2	−0.194	0.227	−0.170	0.288
T	0.250	0.119	0.101	0.527
N	0.614	<0.001	0.458	0.004
G	0.387	0.016	0.443	0.006
CK19 (by IHC)	0.625	<0.001	0.448	0.005
MMP-9	0.898	<0.001	×	×
CK19 mRNA	×	×	0.898	<0.001

## References

[1] Veronesi U., Paganelli G., Galimberti V., Viale G., Zurrida S., Bedoni M., Costa A., de Cicco C., Geraghty J.G., Luini A. (1997). Sentinel-node biopsy to avoid axillary dissection in breast cancer with clinically negative lymph-nodes. Lancet.

[2] Galimberti V., Cole B.F., Zurrida S., Viale G., Luini A., Veronesi P., Baratella P., Chifu C., Sargenti M., Intra M. (2013). Axillary dissection *versus* no axillary dissection in patients with sentinel-node micrometastases (IBCSG 23-01): A phase 3 randomised controlled trial. Lancet Oncol..

[3] Giuliano A.E., Jones R.C., Brennan M., Statman R. (1997). Sentinel lymphadenectomy in breast cancer. J. Clin. Oncol..

[4] Krag D.N., Anderson S.J., Julian T.B., Brown A.M., Harlow S.P., Costantino J.P., Ashikaga T., Weaver D.L., Mamounas E.P., Jalovec L.M. (2010). Sentinel-lymph-node resection compared with conventional axillary-lymph-node dissection in clinically node-negative patients with breast cancer: Overall survival findings from the NSABP B-32 randomised phase 3 trial. Lancet Oncol..

[5] Giuliano A.E., Kirgan D.M., Guenther J.M., Morton D.L. (1994). Lymphatic mapping and sentinel lymphadenectomy for breast cancer. Ann. Surg..

[6] Viale G., Bosari S., Mazzarol G., Galimberti V., Luini A., Veronesi P., Paganelli G., Bedoni M., Orvieto E. (1999). Intraoperative examination of axillary sentinel lymph nodes in breast carcinoma patients. Cancer.

[7] Tsujimoto M., Nakabayashi K., Yoshidome K., Kaneko T., Iwase T., Akiyama F., Kato Y., Tsuda H., Ueda S., Sato K. (2007). One-step nucleic acid amplification for intraoperative detection of lymph node metastasis in breast cancer patients. Clin. Cancer Res..

[8] Osako T., Iwase T., Kimura K., Yamashita K., Horii R., Yanagisawa A., Akiyama F. (2011). Intraoperative molecular assay for sentinel lymph node metastases in early stage breast cancer: A comparative analysis between one-step nucleic acid amplification whole node assay and routine frozen section histology. Cancer.

[9] Sagara Y., Ohi Y., Matsukata A., Yotsumoto D., Baba S., Tamada S., Sagara Y., Matsuyama Y., Ando M., Rai Y. (2013). Clinical application of the one-step acid amplification method to detect sentinel node metastases in breast cancer. Breast Cancer.

[10] Guillen-Paredes M.P., Carrasco-González L., Cháves-Benito A., Campillo-Soto A., Carrillo A., Aguayo-Albasini J.L. (2011). One-step nucleic acid amplification (OSNA) assay for sentinel lymph node metastases as an alternative to conventional postoperative histology in breast cancer: A cost-benefit analysis. Cir. Esp..

[11] Classe J.M., Baffert S., Sigal-Zafrani B., Fall M., Rousseau C., Alran S., Rouanet P., Belichard C., Mignotte H., Ferron G. (2012). Cost comparison of axillary sentinel lymph node detection and axillary lymphadenectomy in early breast cancer. A national study based on a prospective multi-institutional series of 985 patients “on behalf of the group of surgeons from the French Unicancer Federation”. Ann. Oncol..

[12] Lohrisch C., Paltiel C., Gelmon K., Speers C., Taylor S., Barnett J., Olivotto I.A. (2006). Impact on survival of time from definitive surgery to initiation of adjuvant chemotherapy for early-stage breast cancer. J. Clin. Oncol..

[13] Jung S.Y., Sereika S.M., Linkov F., Brufsky A., Weissfeld J.L., Rosenzweig M. (2011). The effect of delays in treatment for breast cancer metastasis on survival. Breast Cancer Res. Treat..

[14] Gainer S.M., Hunt K.K., Beitsch P., Caudle A.S., Mittendorf E.A., Lucci A. (2012). Changing behavior in clinical practice in response to the ACOSOG Z0011 trial: A survey of the American Society of Breast Surgeons. Ann. Surg. Oncol..

[15] Song Z.B., Ni J.S., Wu P., Bao Y.L., Liu T., Li M., Fan C., Zhang W.J., Sun L.G., Huang Y.X. (2015). Testes-specific protease 50 promotes cell invasion and metastasis by increasing NF-κB-dependent matrix metalloproteinase-9 expression. Cell Death Dis..

[16] Bottino J., Gelaleti G.B., Maschio L.B., Jardim-Perassi B.V., de Campos Zuccari D.A. (2014). Immunoexpression of ROCK-1 and MMP-9 as prognostic markers in breast cancer. Acta Histochem..

[17] Mylona E., Nomikos A., Magkou C., Kamberou M., Papassideri I., Keramopoulos A., Nakopoulou L. (2007). The clinicopathological and prognostic significance of membrane type 1 matrix metalloproteinase (MT1-MMP) and MMP-9 according to their localization in invasive breast carcinoma. Histopathology.

[18] Ranogajec I., Jakic-Razumovic J., Puzovic V., Gabrilovac J. (2012). Prognostic value of matrix metalloproteinase-2 (MMP-2), matrix metalloproteinase-9 (MMP-9) and aminopeptidase N/CD13 in breast cancer patients. Med. Oncol..

[19] Sung H., Choi J.Y., Lee S.A., Lee K.M., Han S., Jeon S., Song M., Lee Y., Park S.K., Yoo K.Y. (2012). The association between the preoperative serum levels of lipocalin-2 and matrix metalloproteinase-9 (MMP-9) and prognosis of breast cancer. BMC Cancer.

[20] Zeng Y., Liu C., Dong B., Li Y., Jiang B., Xu Y., Meng L., Wu J., Qu L., Shou C. (2013). Inverse correlation between Naa10p and MMP-9 expression and the combined prognostic value in breast cancer patients. Med. Oncol..

[21] Zhao S., Ma W., Zhang M., Tang D., Shi Q., Xu S., Zhang X., Liu Y., Song Y., Liu L. (2013). High expression of CD147 and MMP-9 is correlated with poor prognosis of triple-negative breast cancer (TNBC) patients. Med. Oncol..

[22] Santaballa A., de La Cueva H., Salvador C., García-Martínez A.M., Guarín M.J., Lorente D., Palomar L., Aznar I., Dobón F., Bello P. (2013). Advantages of one step nucleic acid amplification (OSNA) whole node assay in sentinel lymph node (SLN) analysis in breast cancer. SpringerPlus.

[23] Tamaki Y., Sato N., Homma K., Takabatake D., Nishimura R., Tsujimoto M., Yoshidome K., Tsuda H., Kinoshita T., Kato H. (2012). Routine clinical use of the one-step nucleic acid amplification assay for detection of sentinel lymph node metastases in breast cancer patients: Results of a multicenter study in Japan. Cancer.

[24] Ruano M.A., Lopez-Bonet E., Buxó M., Tuca-Rodríguez F., Vila-Camps E., Alvarez E., Martin-Castillo B., Menendez J.A. (2014). An improved axillary staging system using the OSNA assay does not modify the therapeutic management of breast cancer patients. Sci. Rep..

[25] Osako T., Tsuda H., Horii R., Iwase T., Yamauchi H., Yagata H., Tsugawa K., Suzuki K., Kinoshita T., Akiyama F. (2013). Molecular detection of lymph node metastasis in breast cancer patients treated with preoperative systemic chemotherapy: A prospective multicentre trial using the one-step nucleic acid amplification assay. Br. J. Cancer.

[26] Ohi Y., Umekita Y., Sagara Y., Rai Y., Yotsumoto D., Matsukata A., Baba S., Tamada S., Matsuyama Y., Ando M. (2012). Whole sentinel lymph node analysis by a molecular assay predicts axillary node status in breast cancer. Br. J. Cancer.

[27] Heo D.S., Choi H., Yeom M.Y., Song B.J., Oh S.J. (2014). Serum levels of matrix metalloproteinase-9 predict lymph node metastasis in breast cancer patients. Oncol. Rep..

[28] Decock J., Hendrickx W., Wildiers H., Christiaens M.R., Neven P., Drijkoningen M., Paridaens R. (2005). Plasma gelatinase levels in patients with primary breast cancer in relation to axillary lymph node status, Her2/neu expression and other clinicopathological variables. Clin. Exp. Metastasis.

[29] Pellikainen J.M., Ropponen K.M., Kataja V.V., Kellokoski J.K., Eskelinen M.J., Kosma V.M. (2004). Expression of matrix metalloproteinase (MMP)-2 and MMP-9 in breast cancer with a special reference to activator protein-2, HER2, and prognosis. Clin. Cancer Res..

[30] Wu Q.W., Yang Q.M., Huang Y.F., She H.Q., Liang J., Yang Q.L., Zhang Z.M. (2014). Expression and clinical significance of matrix metalloproteinase-9 in lymphatic invasiveness and metastasis of breast cancer. PLoS ONE.

[31] Tang D., Piao Y., Zhao S., Mu X., Li S., Ma W., Song Y., Wang J., Zhao W., Zhang Q. (2014). Expression and correlation of matrix metalloproteinase-9 and heparanase in patients with breast cancer. Med. Oncol..

[32] Sullu Y., Demirag G.G., Yildirim A., Karagoz F., Kandemir B. (2011). Matrix metalloproteinase-2 (MMP-2) and MMP-9 expression in invasive ductal carcinoma of the breast. Pathol. Res. Pract..

[33] Huang J., Ang L., Liu M.Q., Hu H.G., Wang J., Zou Q., Zhao Y., Zheng L., Zhao M., Wu Z.S. (2014). Serum and tissue expression of gelatinase and Twist in breast cancer. Eur. Rev. Med. Pharmacol. Sci..

[34] Sobin L.H., Gospodarowicz M.K., Wittekind C. (2009). UICC TNM Classification of Malignant Tumours.

[35] Wolff A.C., Hammond M.E., Hicks D.G., Dowsett M., McShane L.M., Allison K.H., Allred D.C., Bartlett J.M., Bilous M., Fitzgibbons P. (2013). Recommendations for human epidermal growth factor receptor 2 testing in breast cancer: American Society of Clinical Oncology/College of American Pathologists clinical practice guideline update. J. Clin. Oncol..

[36] Allred D.C., Harvey J.M., Berardo M., Clark G.M. (1998). Prognostic and predictive factors in breast cancer by immunohistochemical analysis. Mod. Pathol..

